# Editorial: Emerging mechanisms in neurodegenerative disease pathogenesis: vertebrate and invertebrate model organisms

**DOI:** 10.3389/fnmol.2026.1891031

**Published:** 2026-06-17

**Authors:** Sandra Almeida, Yuyu Song, Filipe Marques, Nidhi Sharma

**Affiliations:** 1 Department of Neurology, University of Massachusetts Chan Medical School, Worcester, MA, United States; 2 Department of Neurology, Harvard Medical School and Massachusetts General Hospital, Boston, MA, United States; 3 Department of Neurobiology, University of Chicago, Chicago, IL, United States

**Keywords:** Alzheimer's disease, amyotrophic lateral sclerosis, axonal transport, myogranules, neuroinflammation, Parkinson's disease, protein aggregation, RNA metabolism

Neurodegenerative diseases (NDDs), including Alzheimer's disease (AD), Parkinson's disease (PD), and Amyotrophic Lateral Sclerosis (ALS) remain among the most therapeutically intractable conditions and impose an escalating global burden. Despite decades of research, disease-modifying treatments remain limited, revealing a fundamental gap between mechanistic discovery and translational success. Bridging this gap demands not only the identification of novel molecular targets, but also experimental systems capable of capturing disease-relevant pathology with sufficient mechanistic resolution.

This Research Topic highlights how diverse model systems can be leveraged to elucidate pathogenic mechanisms across NDDs. The collection includes three original research articles and one mini-review, spanning invertebrate models, transgenic mice, and human neuronal cell systems. Together, these studies demonstrate how simplified and genetically tractable models can reveal mechanistic interactions that are often obscured in complex mammalian disease contexts, while also providing platforms for the evaluation of emerging therapeutic strategies.

The study by Chakraborty et al. investigates the functional relationship between two major PD-associated proteins, leucine-rich repeat kinase 2 (LRRK2) and α-synuclein (α-syn), during axonal transport. Using *Drosophila* as a model, the authors show that although LRRK2 does not significantly disrupt cargo movement, LRRK2 overexpression induces modest transport defects, suggesting a modulatory role rather than an essential role in transport regulation. Notably, specific PD-associated mutations in LRRK2 differentially influence α-syn-mediated transport impairments, revealing domain-specific effects on vesicle dynamics. Strikingly, co-expression of LRRK2 alleviated α-syn-induced transport defects and reduced vesicle stalling independently of neuronal cell death, indicating that LRRK2 selectively modulates transport dynamics through mechanisms distinct from its kinase-dependent activity. This study highlights the power of *Drosophila* genetics for resolving complex protein interactions underlying PD-related neuronal dysfunction.

While the PD study focuses on intracellular transport dynamics, the focus shifts to toxic protein aggregation in AD in the study by De Plano et al., which explores the potential protective effect of a bacteriophage identified through phage display technology against amyloid-beta (Aβ) toxicity. Aβ peptides accumulate in the brain and form amyloid plaques in AD, the most common neurodegenerative disorder. Among Aβ species, soluble oligomeric aggregates are considered particularly toxic, as they are strongly associated with synaptic dysfunction, oxidative and metabolic stress, and correlate more closely with cognitive decline than plaque burden. In this study, the bacteriophage 12CIII1 was shown to preferentially bind Aβ oligomers, with reduced affinity for Aβ monomers and small oligomers, suggesting a capacity to selectively target toxic Aβ species. Consistent with this selectivity, preincubation with 12CIII1 protected differentiated SH-SY5Y neuroblastoma cells from Aβ42-induced neurite shortening, loss of cell viability, and transcriptional changes consistent with mitochondrial dysfunction and oxidative stress. Together, these findings support a role for metabolic impairment in AD-related neurodegeneration and identify 12CIII1 as a promising phage-based strategy to selectively target higher-order Aβ oligomeric assemblies.

Complementing this focus on toxic protein species, Ham et al. expand the discussion toward neuroinflammation, a major pathological process increasingly recognized as a central driver of AD progression. The study leverages the Tg2576 transgenic mouse model that overexpresses a human amyloid precursor protein (APP) with the Swedish mutation, alongside Aβ-stimulated BV-2 murine microglial cells to probe neuroinflammation as a central driver of AD pathology. This model enables simultaneous evaluation of both amyloid burden and neuroinflammatory responses in a genetically defined context. This study demonstrates that Chitinase-3-like 1 (CHI3L1), a secreted glycoprotein which is upregulated in AD, could be a promising therapeutic target. A novel CHI3L1 monoclonal antibody, H1, was shown to cross the blood–brain barrier and, upon administration, alleviated memory impairment while reducing amyloid deposition. Mechanistically, H1 inhibited the extracellular signal-regulated kinase (ERK) and nuclear factor-kB (NF-κB) signaling pathways and suppressed pro-inflammatory microglial marker expression, results further confirmed by proteomic and gene expression analysis. Together, these findings position CHI3L1 neutralization as a promising immunotherapeutic strategy for attenuating neuroinflammation in AD.

Broadening the perspective from protein aggregation and neuroinflammation to RNA metabolism and tissue vulnerability, the final contribution explores how stress-associated ribonucleoprotein assemblies may contribute to ALS pathogenesis. The mini-review by Ishaq and Russell provides a timely synthesis of how TDP-43-associated ribonucleoprotein assemblies may connect motor neuron degeneration with skeletal muscle pathology in ALS. This review bridges stress granules in neural tissues with myogranules in regenerating muscle, positioning these dynamic RNA-protein condensates not simply as bystanders of cellular stress but as potential nodes where impaired RNA metabolism, defective proteostasis, cytoplasmic TDP-43 accumulation, and tissue vulnerability converge. The authors emphasize that granule formation may be protective, compensatory, or pathological depending on context, duration, composition, and clearance, and avoid overstating liquid-liquid phase separation as a universal causal mechanism. While the evidence directly linking myogranules to ALS remains elusive and largely correlative, this review highlights the importance of considering ALS pathogenesis through integrated neuron-muscle interactions rather than solely neuron-intrinsic mechanisms.

The articles included in this Research Topic collectively employ diverse model systems—from *Drosophila* and transgenic mice to differentiated human neuronal cell lines, to investigate emerging mechanisms of neurodegeneration across molecular, cellular, and organismal scales. Together, these studies provide new insights into multiple aspects of pathogenesis, including domain-specific protein interactions in PD, amyloid aggregation toxicity and neuroinflammation in AD, and TDP-43-associated ribonucleoprotein assemblies in ALS. In addition, several contributions explore therapeutic strategies aimed at selectively interfering with toxic protein species and downstream pathogenic mechanisms. Importantly, this Research Topic highlights how simplified and genetically tractable systems can reveal causal mechanisms that are often difficult to resolve in complex mammalian disease contexts. As NDDs increasingly emerge as multifactorial diseases driven by interconnected pathways across diverse cell types and tissues, integrating insights across different biological systems will be critical not only for advancing mechanistic understanding, but also for accelerating the development of effective therapeutic strategies.

